# Effects of In Ovo Taurine Injection on Embryo Development, Antioxidant Status, and Bioactive Peptide Content in Chicken Embryos (*Gallus gallus domesticus*)

**DOI:** 10.3390/ijms252111783

**Published:** 2024-11-02

**Authors:** Monika Łukasiewicz Mierzejewska, Marta Kotuszewska, Kamila Puppel, Beata Madras Majewska

**Affiliations:** 1 Institute of Animal Science, Warsaw University of Life Sciences, Ciszewskiego 8 Street, 02-786 Warsaw, Poland; kamila_puppel@sggw.edu.pl (K.P.); beata_madras_majewska@sggw.edu.pl (B.M.M.); 2 Scientific Circle “Aves”, Warsaw University of Life Sciences, Ciszewskiego 8 Street, 02-786 Warsaw, Poland; marta.kotuszewska@gmail.com

**Keywords:** in ovo, taurine, embryo development, antioxidant status, peptides

## Abstract

Stress in birds disrupts the homeostasis of the organism, leading to an inability to neutralize reactive oxygen species. Taurine, an effective antioxidant, affects various cellular mechanisms, including cation modulation, protein phosphorylation, and cell proliferation. The aim of the study was to evaluate the effect of colloid with taurine applied in ovo to Albumin on embryonic development, oxidative stress indicators and the content of bioactive peptides—carnosine and anserine—in the pectoral muscle. The research materials were eggs of the parent flock (Ross 308) divided into four groups (K (without injection), T50-concentration of taurine hydrocolloid 50 ppm (mg/L); T100-colloid concentration 100 ppm (mg/L) taurine; T500-colloid concentration of 500 ppm (mg/L) taurine). The experimental solutions were injected in an amount of 0.3 mL into egg white. Eggs were incubated under standard incubation conditions. The addition of 100 and 500 ppm taurine had a highly significant (*p* = 0.001) effect on the plasma antioxidant potential in chicks. The level of anserine increased with increasing concentrations of taurine. These changes were highly significant (*p* = 0.007). The level of anserine in the T2 and T3 groups was determined to be 2.5 times higher than in the pectoral muscles of embryos not treated with taurine colloid. An analysis of the results showed that the administration of an increased dose of hydrocolloid with taurine increased the content of carnosine and anserine in the pectoral muscle. Colloid with taurine applied in ovo to chicken white egg reduces oxidative stress and increases homeostasis of the organism.

## 1. Introduction

Taurine (2-aminoethanesulfonic acid) is a non-protein sulfuric amino acid. Due to the replacement of the carboxylic group with the sulfonic group, it is not possible to form a peptide bond, so the amino acid does not form proteins. It occurs in the free state in the body and represents nearly 90% of all free amino acids in the cytosol [1,2]. The presence of the sulfonic group makes taurine an amino acid of the highest acidic character [2,3,4,5].

Taurine plays a critical role in several physiological processes, including the stabilization of cell membranes, the conjugation of bile acids, the maintenance of calcium balance, detoxification, reduction in inflammation, and modulation of the immune response [6]. While taurine is not categorized as an essential amino acid for poultry due to its endogenous synthesis [7], it has been suggested that the demand for taurine may rise in birds facing different stressors [8]. Previous research indicates that increased taurine intake can alleviate stress related to heat, immunological challenges, and high stocking densities in poultry [6]. Additionally, studies have shown that taurine supplementation can enhance productivity in broiler chickens, improving both weight gain and feed efficiency [9]. Furthermore, the addition of taurine to diets has been associated with a reduction in oxidative stress, achieved through the activation of antioxidant defense mechanisms in these birds [10]. Nonetheless, there is a considerable lack of research on the impact of dietary taurine on laying hens.

Taurine concentration in most cells is quite high, especially in the retina, kidney core and brain [2,11]. It is responsible for maintaining isosomy—a constant osmotic pressure [2]. As an organic osmolyte, it protects the cell from excessive stretching when osmotic imbalances between the cell and body fluid occur by modifying ion levels. Intracellular taurine levels increase in response to increasing osmotic pressure, while they decrease during hypoosmotic stress [11,12]. Revealing its properties during active participation in osmotic regulation, taurine influences such cellular mechanisms as cationic modulation, protein phosphorylation and cell proliferation [12].

Taurine deficiency results in a decrease in the activity of the complex I of the respiratory chain called NADH dehydrogenase and a simultaneous increase in the NADH/NAD+ ratio and in mitochondrial NADH. This leads to the inactivation of enzymes participating in the Krebs cycle, such as α-ketoglutarate dehydrogenase, isocitrate dehydrogenase and citrate synthase. These factors interfere with energy metabolism [5,13]. A slowed oxygen metabolism contributes to mild heart failure. Although a healthy heart muscle prefers fatty acids as the primary substrate in the energy metabolism, after a minor heart failure, the metabolism changes in favor of glucose. This is because the level of the transcription factor PPARα responsible for regulating the expression of proteins involved in the metabolism of fatty acids is subject to a decrease [14,15]. Unfortunately, the mitochondria in inefficient heart cells are damaged, which prevents the efficient functioning of glucose metabolic pathways in the aerobic way. Anaerobic glucose metabolism increases, and damaged mitochondria reduce the oxidation of this substrate. Finally, the energy state of the heart does not improve [13,16]. Taurine activates NADH dehydrogenase and enzymes sensitive to it and reduces the NADH/NAD+ ratio during glycolysis. Moreover, it restores the oxidation of fatty acids by increasing the level of PPARα [13].

Taurine is an effective antioxidant. This amino acid can react with highly oxidizing compounds. These include hypochlorous acid. It is formed during a “respiratory burst” in phagocytic neutrophils. H_2_O_2_ together with myeloperoxidase contributes to the oxidation of chloride anions present in the environment in physiological concentrations. They are converted to hypochlorous acid—a cidal agent of neutrophils, also dangerous for the nearest cell structures and even genetic material. Taurine reacts with hypochlorous acid to form taurine chloramine, which is less toxic, and has bactericidal and bacteriostatic properties [2,17,18]. Taurine reduces peroxide production by mitochondria [19]. This is the effect of the formation of conjugates with the residue of tRNALeu uridine, which after coupling is in the Wobble position on the anticodon. Therefore, the interaction of the tRNA AAU anticodon (UUR) with the UUG codon of mitochondrial mRNA becomes more efficient. When the level of taurine is too low to form conjugates, it is not possible to obtain the right amount of proteins and thus enzymes that activate NADH in the respiratory chain. Therefore, there is a production of peroxides that can lead to mitochondrial oxidative stress, and consequently even their apoptosis [20]. Taurine is therefore a source of substrate for the coupling reaction, thanks to which the biosynthesis of mitochondrial proteins is normal, the mitochondria function more efficiently, and the production of peroxides is lower [21,22].

Chick embryos (*Gallus gallus domesticus*) are one of the best studied biological organisms. Their development and growth takes place outside the mother’s body, which facilitates a wide variety of experiments [23]. The short incubation period (only 21 days) as well as the availability, low price and less controversial consequences compared to experiments on rodents and large animals make chicken embryos an ideal research model.

The aim of this study was to evaluate the effect of water colloid with taurine applied in ovo to hen’s egg white on embryonic development, oxidative stress indicators and the contents of bioactive peptides—carnosine and anserine—in the pectoral muscle.

## 2. Results and Discussion

The chicken embryos used in this study are among the best-studied biological organisms [24]. They are characterized by many features that allow researchers to perform a variety of experiments and studies. The development and growth of the embryo takes place outside the mother’s body, in the egg. This is ensured during short-term incubation (only 21 days). These two factors make it easier to trace the embryogenesis and raise the possibility to manipulate this process, for example, through the intervention of certain substances in ovo. The condition for proper development is to ensure proper temperature, humidity and egg rotation during incubation [24,25]. Additional arguments for the justified use of chicken embryos as research models include: easy access, low price, and less controversial effects on the living organism compared to experiments on rodents and large animals [26].

Taurine acts as an antioxidant, as evidenced by results showing superoxide dismutase (SOD) levels. The injection of taurine in ovo increased serum SOD levels in embryos, but the differences were not statistically significant (*p* = 0.265). Higher taurine concentrations resulted in higher levels of superoxide dismutase. Analysis of the results shows that the administration of an increased dose of taurine colloid increased carnosine and anserine content in pectoral muscles. Anserine levels increased with increasing taurine concentration, and these changes were highly significant (*p* = 0.007).

Table 1 shows the results of mortality and fertilized eggs used for further studies. The exposure of embryos to taurine on the first day of development caused an increase in embryo mortality. The highest mortality was recorded in group T2. Berning et al. [27] also received unfavorable results for embryo survival rate after administration of taurine in ovo to hen eggs on the first three days of development. The introduction of taurine into eggs can lead to an excessive stress response in the bodies of developing embryos. High concentrations of taurine can upset the metabolic balance, resulting in increased mortality. In addition, taurine in excess may affect the development of the nervous system or other key systems, which may contribute to higher mortality. Increased taurine levels can affect cellular function and egg cell metabolism. High taurine concentrations can interfere with processes involved in egg cell maturation, leading to reduced egg quality. This may result in a reduced fertilization capacity, which explains the lower percentage of fertilized eggs.

Negative effects of taurine hydrocolloid on the body weight of embryos after hatching were also observed. On average, the embryos with applied taurine at a concentration of 500 ppm (T3) were 1.3 g lighter. Liu et al. [28] sought to determine the effect of taurine on rat embryos by supplementing it in pregnant females (300 mg/kg/day from the 12th day after fertilization to delivery), demonstrated that it reduces body weight in newborn rodents.

### 2.1. Taurine Effect on Oxidative Stress Indicators

Oxygen, an essential element for life, becomes a toxic radical due to certain biochemical reactions in the body. During processes such as mitochondrial respiratory chain, microsomal electron transport chain, the autooxidation of biologically active compounds or some enzymatic reactions, molecular oxygen O_2_ is not completely reduced. Reactive oxygen species (ROS) are thus formed, characterized by the presence of at least one unpaired electron on the valence coating, e.g., O_2_^•−^ superoxide anion radical or HO_2_^•^ hydroperoxide radical. The presence of these chemical individuals is not desirable; therefore, the organism must neutralize them with antioxidants [29]. This ability is referred to as antioxidant potential. In the state of homeostasis of the organism, the production and neutralization of reactive oxygen species is strictly controlled. The consequence of this imbalance is oxidative stress. It is caused by an increased formation of ROS or due to insufficient amount of antioxidants [30]. The body is equipped with protective mechanisms against oxidative stress. The first line of defense consists in breaking the chains of oxidation reaction thanks to antioxidant enzymes, such as superoxide dismutase, catalase (CAT) and peroxidase, which catalyze reactions with free radicals. Non-enzymatic antioxidants form the second line of defense. These are low-molecular compounds such as vitamins (ascorbic acid), microelements (Zn, Se), metal ion-binding proteins (ferritin), thiol group compounds (glutathione, coenzyme A), flavonoids, or lipoic acid. They deactivate free radicals that have not been neutralized by enzymes. The third line of defense includes all repair processes needed to eliminate damage caused by free radicals [29,31]. When the body cells are exposed to long-term oxidative stress, organic compounds such as lipids, proteins and nucleic acids are damaged. The effect of the disturbed oxidative potential is visible on livestock farms in the form of poorer production results (daily weight gain, feed use, etc.) and poorer-quality animal products. Maintaining a proper general health status prevents the induction of reactive oxygen species. Research shows that farm animals are exposed to oxidative stress in the following situations: pregnancy, perinatal period, inadequately balanced ration, diseases and infections, physical exertion and temperature changes (heat stress) [29].

In our study, TAS (total antioxidant status) was determined. It is one of several indirect methods of free radicals’ measurement. By spectrometry, the level of substrates or products of antioxidant reactions is estimated. In determining TAS, peroxidase catalyzes in the reaction mixture the oxidation of ABTS by hydrogen peroxide to the cation radical ABTS+, which has a blue-green coloration. The more antioxidants there are in the environment, the slower and weaker the coloration of the sample. Spectrometric detection after an inhibition of the mixture’s greening is used to determine the level of antioxidants in the tested solution [32]. In addition to TAS, we can distinguish oxygen radical absorbance (ORAC), antioxidant capacity equivalent (TEAC), the ability of antioxidants to reduce iron ions (Fe^3+^) to iron ions (Fe^2+^) (FRAP), the concentration of oxidation product over time, the point at which antioxidants cease to act (TRAP), the concentration of vitamins A, C and E in plasma, and the redox reaction based on the concentration of substrates or products of free radical reactions (aldehydes, e.g., malonic dialdehyde (MDA) and TBA test (thiobarbituric acid)) [29].

In the study on hen embryos, the lower TAS (mmol/L) level in the T2 group (100 ppm taurine) compared to the control group and the lowest TAS level in the T3 group (500 ppm taurine) compared to the other experimental groups were determined. The addition of 100 and 500 ppm taurine had a highly significant (*p* = 0.001) effect on the plasma antioxidant potential (the lower the TAS value, the higher the antioxidant level) (Table 2).

This is also confirmed by the results showing the level of superoxide dismutase (SOD) (Table 2), the most effective enzyme included in the first line of defense against oxidative stress [29]. It catalyzes the decomposition of superoxide radicals into hydrogen peroxide and oxygen [29,32]. No significant changes were observed in peroxide dismutase (U/mL) serum levels in embryos treated with taurine, but the in ovo injection of this amino acid increased the level (*p* = 0.265). The higher the taurine concentration, the higher the level of superoxide dismutase.

Taurine therefore acts as an antioxidant. Shivananjappa and Muralidhara [33] proved the antioxidant effects of taurine in rat studies. An experiment was conducted in which six study groups were set up composed of pregnant female rats, half of which had streptosotcocine-induced diabetes on the fourth day of pregnancy. By supplementing pregnant females with taurine in feed at doses of 0.5 or 1 g taurine/1 kg b.w. from the 5th to 12th day of pregnancy (except for the control group and one group of rats with diabetes mellitus), the tendency of this amino acid to weaken oxidative stress was studied. Blood was collected, and liver and embryos were obtained from rats slaughtered on the 20th day of pregnancy. Oxidative stress markers (RO (reactive oxygen species), MDA, CAT, SOD, GR glutathione reductase and GPx glutathione peroxidase, GST glutathione transferase) were determined in embryos and the mother’s liver. Although taurine had a beneficial effect on oxidative indices in the mother liver, the results did not show significant differences in non-diabetic females. On the other hand, the induction of diabetes contributed to an increase in ROS and MDA, and a high level of taurine supplementation significantly lowered these markers. A similar protective effect of taurine in alleviating the reduction in antioxidants was observed by measuring the activity of enzymes. SOD and GR concentrations were normalized (a clear decrease in the level of these enzymes was observed in diabetic mothers without supplementation) (*p* < 0.05). ROS concentration in embryos was obtained from mothers with diabetes. While the high dose of taurine supplementation did not significantly affect the ROS concentration in embryos from healthy mothers, it significantly (*p* < 0.05) lowered the elevated ROS level in embryos from diabetic mothers. The same relationship was observed in the case of enzymatic antioxidants—embryos from healthy mothers were characterized by insignificant statistical differences after the use of taurine, while a high dose of taurine significantly increased the concentrations of enzymes SOD, CAT, GPx, GR and GST in embryos obtained from diabetic females. Similarly, Winiarska et al. [34] treated rabbits suffering from diabetes with taurine (1% taurine in drinking water for 3 weeks), and observed a reduction in reactive oxygen species caused by alloxan. Moreover, the treated animals were characterized by an increased activity of CAT, SOD and GR.

Many studies have focused on the protective effect of taurine against toxicity caused by various factors. The team of Tas et al. [35] investigated the effect of taurine supplementation administered with water on oxidative stress in experimental thyroid hypothyroidism in rats. The results show a highly significant effect of taurine on increasing glutathione (antioxidant) levels in the liver compared to the control group. A statistically significant difference in this marker was also observed in the kidney. The measurement of malondialdehyde in plasma also indicated the antioxidant effect of taurine. The plasma obtained from rats supplemented with taurine was characterized by a highly significantly lower MDA level compared to the control group rodents (*p* < 0.001). Other studies documented a reduction in ROS production while increasing the activity of antioxidant enzymes SOD, CAT or GPx after taurine application, i.e., to mouse hepatocytes in which oxidative stress was induced by fluorine (with NaF) [36]; to rat vascular smooth muscle cells in which oxidative stress was induced by homocysteine [37]; to mouse melanoma cells B16F10 [38]. All these studies, accompanied by our study, show that taurine antagonizes the effects of many factors responsible for oxidative stress generation on the production of reactive oxygen species and antioxidative enzyme activity. It is also worth mentioning that mammalian embryos can capture taurine from their own environment [39].

### 2.2. Taurine Effect on Bioactive Peptides Content in Tissues

Analysis of the results shows that the administration of an increased dose of hydrocolloid with taurine increased the content of carnosine and anserine in the pectoral muscle (Table 3). The level of anserine increased with increasing concentrations of taurine. These changes were highly significant (*p* = 0.007). The level of anserine in the T2 (100 ppm) and T3 (500 ppm) groups was determined to be 2.5 times higher than in the pectoral muscles of embryos not treated with taurine colloid. Slightly lower, but still with an upward trend, carnosine content in the examined tissue was determined, with these differences being statistically insignificant (*p* = 0.495). The dipeptides mentioned above were the subject of the study because of their pro-healthy properties resulting from their increased content in the diet.

Anserine is a methylated metabolite of carnosine. Both these organic compounds are histidine dipeptides performing similar, very important functions. Carnosine occurs in the skeletal muscles of mammals and birds, while anserine is only found in animal muscles [40]. Carnosine exhibits its antioxidant effects by donating the H^+^ ion to neutralize free radicals and by creating a transfer that carries free radicals and harmful aldehydes [41,42]. In addition, carnosine buffers pH, is responsible for the activation of muscle ATPase, chelates copper, zinc and iron ions, protects against lipid peroxidation and protein oxidation, and has an anti-glycation effect [40,43]. This dipeptide also affects the integrity of the DNA molecule and increases the acetylation of histones [44,45]. It is responsible for the homeostasis of calcium ions in skeletal muscles, stimulates the activity of phosphorylase inducing the breakdown of glycogen, and modulates the strength of muscle spasms [40]. Carnosine induces cancer cell apoptosis [46]. It prevents coronary heart disease by lowering blood pressure [47]. Carnosine is also beneficial for the mitigation of cell-aging effects [40]. Anserine, like carnosine, maintains an acid–base balance, has an antioxidant effect, modulates muscle contractility and maintains the trans-membrane potential [40,43]. Studies on animal models have shown a beneficial effect of anserine on the smooth functioning of the nervous and circulatory systems and kidney function [48,49,50]. Anserine, unlike carnosine, does not chelate copper ions [51]. However, it has the ability to regulate the action of carnosine in the body [40].

Carnosine (β-alanyl-L-histidine) and anserine (β-alanyl-1-methyl-L-histidine) are dipeptides with β-alanine and L-histidine in their chemical composition. Studies on increasing the concentration of these two peptides in the pectoral muscles were therefore based mainly on the enrichment of the diet with β-alanine or L-histidine. Tomonaga et al. [52] verified the effect of β-alanine. Broilers were given oral β-alanine (0.176; 0.88; 4.4 and 22 mmol/10 mL of water/kg) twice daily for 5 days from day two. Carnosine levels increased depending on the dose (in the pectoral muscle, plasma and brain), but no changes in anserine concentration were observed. Łukasiewicz et al. [53] demonstrated a significant effect of β-alanine supplementation on the anserine content in the pectoral muscles and on the carnosine content in the leg muscles of chicken broilers. Also, Qi et al. [54] observed a square increase in the concentration of carnosine in the pectoral muscles of chicken broilers after supplementation with β-alanine (0, 250, 500, 1000, 2000 mg/kg feed) for 42 days. The influence of histidine was addressed by Haug et al. [55]. During their research, 1 g of histidine per kg of chicken broiler feed was supplemented. This resulted in an increase in carnosine level by 64% and anserine level by 10% in the pectoral muscle. In turn, Kai et al. [56] supplemented 14-day-old female chicken broilers with three different levels of histidine for 10 days: in short supply (67%), covering 100% of the histidine requirement, and in excess (200%). The reduced dose resulted in undetectable levels of carnosine in the pectoral muscles, while the increased dose significantly increased the peptide concentration. Similar, but lower, results were obtained for anserine. Kralik et al. [57] conducted a wider study combining L-histidine and β-alanine in feed supplementation for the last 3 weeks of fattening. A diet supplemented with β-alanine (0; 05; 1%) ensured a 20.48% increase in carnosine content in the pectoral muscle at a 1% dose. On the other hand, supplementation with L-histidine (0; 0.3; 0.5%) increased the level of carnosine by 25.96% in the same muscles at the highest concentration of administered amino acid. Rats were also supplemented with histidine. Their diet was enriched with histidine in excess, or a dose of this amino acid was given with deficiency. The histidine deficiency caused a decrease in the concentration of mainly carnosine, but also of anserine in the gastrocyaneus muscle, while the excess doubled their concentration [58]. β-alanine (100 mg/kg b.w.) and L-histidine (12.5 mg/kg b.w.) were also supplemented with horse feed for 30 days. The study demonstrated that these two amino acids increase the content of carnosine in the horse gluteus medius muscle [59]. An outstanding observation was made in the study of the effect of blood meal (0; 5; 10%) given to chicken broilers (from the 1st day of life for 5 weeks) on the carnosine and anserine contents of the pectoral muscle. It was shown that the addition of 5% blood meal in the diet of chickens increases the level of carnosine, while the concentration of anserine does not change [60]. The beneficial effect of β-alanine and L-histidine on the increase in carnosine and anserine content in muscles is logical due to the chemical structure of dipeptides. There is little proven influence of other factors that can contribute to similar effects; therefore, our study indicates new possibilities of meat enrichment through taurine with pro-healthy histidine peptides.

## 3. Materials and Methods

The study was carried out on hen embryos (*Gallus gallus domesticus*). The experimental procedures followed the Polish National Legislation. The study material consisted of hatching eggs (n = 180) from the parent flock (Ross 308). Broiler chicken eggs from 39-week-old Ross X Ross 308 hens were obtained from a commercial hatchery and stored in a refrigerator (10 °C) for 1 day before being placed in an incubator. Eggs were divided into four groups (45 eggs per group): K (no injection), T50—taurine hydrocolloid concentration 50 ppm (mg/L); T100—taurine colloid concentration 100 ppm (mg/L); T500—taurine colloid concentration 500 ppm (mg/L). Experimental solutions were applied at an amount of 0.3 mL into egg white by injection using sterile 27-gauge, 20 mm needles. Eggs were incubated under standard incubation conditions in the same incubator. For testing, eggs were disinfected with 0.05% potassium permanganate solution (KMnO_4_), then placed (for 50 s) in a UV KOR lamp with UVC-J series (egg disinfectants). These devices use the C-type ultraviolet (UV-C) range, i.e., radiation with a wavelength of 180 to 280 nm. This has a lethal effect on living organisms, destroying, among others, bacteria, viruses and molds on the shell. After disinfecting and irradiating the eggs, they were labeled, and the shells were cut with a scalpel, and 0.3 mL of the analyzed solution was inserted using the syringe with a disposable needle into the egg white, then the injection sites were sealed with disposable 25 mm hypoallergenic patches (Polopor by 3M Viscoplast, Katowicka, Poland). Eggs were placed in an incubator (ALMD-1N3–7; Waleński, Gostyń, Poland), where they were then incubated in standard conditions (temperature 37.8 °C, humidity 65%, and egg position changed every 1 h by 45°, until the 18th day of incubation) until the 20th day. After that time, the eggs were opened and the embryos were weighed.

### 3.1. Handling with Embryos

The following parameters were evaluated in 20-day-old embryos: mortality, embryo body weight, selected indicators of blood antioxidant status. After decapitation, the blood was taken from the carotid artery of the embryo into glass tubes, which were left at room temperature to form a clot, and then centrifuged for 5 min at 4000 rpm (MPW-350R centrifuge, MPW Med. Instruments, Warsaw, Poland). The obtained serum (transferred to cryovials) was then stored at −80 °C (TV 75 Vestfrost, Warszawa, Poland) for further analyses. The chicks were then dissected and the yolk sac, heart, liver, breast and intestine were weighed in order to measure their development.

### 3.2. Laboratory Analyses

The total antioxidant potential (TAS) was determined in chicken serum using the RANDOX Laboratory diagnostic kit (Antrim, UK)

Total antioxidant potential (TAS) by RANDOX application.

Incubation of ABTS^®^ with peroxidase (methemoglobin) leads to the formation of a radical ABTS + + cation. This substance is blue-green and can be detected at 600 nm. The antioxidants present in the sample reduce the formation of the blue-green color in proportion to their concentration.

SOD was measured by the degree of reaction inhibition with xanthine oxidase and peroxide radicals, respectively.

The levels of dipeptides carnosine and anserine in the pectoral muscles of bird embryos were determined by high-performance liquid chromatography with reverse phase system RP-HPLC Agilent 1100 (Agilent Technologies, Waldbronn, Germany) and Jupiter C18 300A columns (Phenomenex, Torrance, CA, USA). Mobile phase A was a mixture of acetonitrile with water (30:70) and acid 0.1% TFA (both reagents from Merck, Darmstadt, Germany), while phase B was a mixture of acetonitrile with water (70:30) and acid 0.1% TFA. Flow through the column was 1.4 mL/min, with UV detection at 214 nm. Quantitative analysis was performed by means of calibration against standards (carnosine Lot # CBB7948 and anserine Lot # BCBF45160 (Sigma-Aldrich, St. Louis, MO, USA)).

### 3.3. Taurine

In the experiment, C2H7NO3S Lot#BCBJ5953V taurine from Sigma-Aldrich, USA with the highest purity (99%) was used, in the form of white powder, easily soluble in water. The molar mass of the substance used was 125.15 g/mol.

### 3.4. Statistical Analysis of Results

The collected results were statistically elaborated using a one-way analysis of variance using the least squares method (SPSS 23.0 PL). The tables include the mean of the least squares and the standard error of the mean (SE).

## 4. Conclusions

In conclusion, taurine has a comprehensive effect on the hen’s body, promoting growth, health and productive performance. However, its supplementation should be appropriately tailored to the needs of the animals and their rearing conditions for optimal results. Typical taurine doses in poultry farming range from 0.1% to 0.5% of feed dry matter (i.e., 1 to 5 g of taurine per kg of feed). The cost of taurine application and its use in poultry farming can depend on several factors, such as the form of taurine (powder, capsules, feed mixes), the dose, the amount of poultry and the scale of production. Costs are determined by scale of production, i.e., in small farms, taurine costs may seem high per kilogram of feed, but in large industrial farms where feed is prepared in bulk, these costs may be more cost-effective.

In this study, we demonstrated that higher concentrations of taurine significantly increased antioxidant potential and bioactive peptide content, with implications for improving poultry health and meat quality. Our research provides clear and reproducible results, contributing to the understanding of taurine’s role in embryonic development and its potential application in poultry production. However, further research is needed to fully understand the practical applications and long-term effects of taurine supplementation in poultry.

## Figures and Tables

**Table 1:** Hatching parameters.

		Group				SE	*p*-Value
Parameter		K *	T1	T2	T3		
Fertilized eggs [%]		85.6	79.7	75.6	82.1	4.19	0.085
Mortality [%]		22.2	32.2	42.3	35.4	5.45	0.154
Average body weight [g]		49.0	48.3	48.0	48.2	4.50	0.650

* K–control group; T1—taurine colloid concentration 50 ppm; T2—taurine colloid concentration 100 ppm; T3—taurine colloid concentration 500 ppm; SE—standard error of the mean.

**Table 2:** The levels of TAS (mmol/L) and SOD (U/mL) in the serum of hen embryos.

		Group *				SE	*p*-Value
Parameter		K	T1	T2	T3		
TAS (mmol/L)		1.615 ^A,d^	1.866 ^A,B,C^	1.549 ^B^	1.499 ^C,d^	0.080	0.001
SOD (U/mL)		223.74	199.97	238.85	243.45	17.67	0.265

* K—control group; T1—taurine colloid concentration 50 ppm; T2—taurine colloid concentration 100 ppm; T3—taurine colloid concentration 500 ppm; ^A,B,C^—differences statistically significant at *p* ≤ 0.01; ^d^—differences statistically significant at *p* ≤ 0.05; SE—standard error of the mean.

**Table 3:** Content of biologically active peptides in the pectoral muscles of hen embryos (mg/g).

		Group *				SE	*p*-Value
Parameter		K	T1	T2	T3		
Carnosine	Pectoral muscles	0.533	0.68	1.056	1.069	0.321	0.495
Anserine		0.898 ^B^	2.156 ^A^	2.352 ^A^	2.353 ^A^	0.210	0.007

* K—control group; T1—taurine colloid concentration 50 ppm; T2—taurine colloid concentration 100 ppm; T3—taurine colloid concentration 500 ppm; ^A,B^—differences statistically significant at *p* ≤ 0.01; SE—standard error of the mean.

## Data Availability

The data presented in this study are available on reasonable request from the corresponding author.
